# Serum lactate dehydrogenase isoenzyme 1 as a prognostic predictor for metastatic testicular germ cell tumours – reply

**DOI:** 10.1054/bjoc.2000.1450

**Published:** 2000-11

**Authors:** J Shamash

**Affiliations:** Department of Medical Oncology, St. Bartholomew’s Hospital, Ist Floor King George V Building, West Smithfield, London, ECIA 7BE, UK


					1258 Letters to the Editor
British Journal of Cancer (2000) 83(9), 1255-1259 (c) 2000 Cancer Research Campaign
0 500 1000 1500 2000 2500 3000
Days
100
80
60
40
20
0
Survival(%)
100
80
60
40
20
0
0 500 1000 1500 2000 2500
Days
Survival(%)
0
0
500 1000 1500 2000 2500 3000
20
40
60
80
100
Days
Survival
(%)
0 500 1000 1500 2000 2500 3000
Days
100
80
60
40
20
0
Survival(%)
0 500 1000 1500 2000 2500 3000
Days
100
80
60
40
20
0
Survival(%)
0
0
500 1000 1500 2000 2500 3000
20
40
60
80
100
Days
Survival
(%)
Figure 2 (A) The survival for 81 patients with metastatic TGCT according to the initial postsurgical S-LD-1 (---- = 39 patients with S-LD-1 < 112 U L-1
,
- - = 37 with S-LD-1 112-1120 U l-1
, and - . - = five with S-LD-1 > 1120 U l-1
). (B) The survival according to S-hCG (---- = 39 patients with S-hCG < 30 IU l-1
,
- - = 33 with S-hCG 30-4999 IU l-1
, - - - = four with S-hCG 5000-50 000 IU l-1
, - - - = five with S-hCG > 50 000 IU l-1
). (C) The survival according to the risk
groups of the IGCCCG classification (---- = 49 patients with a good prognosis, ---- = 16 with an intermediate prognosis, and - - - = 16 with a poor prognosis).
(D) The survival according to S-AFP (---- = 50 patients with S-AFP < 24 g l-1
, ---- = 26 with S-AFP 24-999 g l-1
, - - - = four with S-AFP 1000-10 000 g l-1
,
- - - = one with S-AFP > 10 000 g l-1
. (E) The survival according to the concomitantly normal and raised levels of S-LD-1 and S-LD (---- = 31 patients with
normal values of S-LD-1 and S-LD, -- -- = eight with a normal value of S-LD-1 and a raised S-LD, and - - - = 42 with a raised S-LD-1 (with or without a raised
S-LD)). (F) The survival according to the S-LD-1/S-LD ratio (---- = 73 patients with an S-LD-1 ratio < 0.375, and - - = eight with a ratio > 0.375)
doi: 10.1054/ bjoc.2000.1450, available online at http://www.idealibrary.com on
Serum lactate dehydrogenase isoenzyme 1 as a
prognostic predictor for metastatic testicular germ cell
tumours - reply
Sir
von Eyben et al describe their experience of splitting total LDH
into its various isoenzymes and have identified LDH isoenzyme 1
(S-LD-1) as having prognostic significance in untreated patients
with metastatic germ cell tumours. The evidence they present
is clearly impressive and S-LD-1 appears to be able to identify
patients with a poor outcome as effectively as the IGCCCG speci-
fication or serum HCG alone.
Letters to the Editor 1259
British Journal of Cancer (2000) 83(9), 1255-1259
(c) 2000 Cancer Research Campaign
We agree that validation of their findings with a larger set of
patients would be useful and also feel that the prognostic role of
S-LD-1 in patients who relapse should also be assessed to see if
its predictive role in this setting is maintained.
J Shamash
Department of Medical Oncology, St. Bartholomew's Hospital, Ist Floor King
George V Building, West Smithfield, London ECIA 7BE, UK

				

